# Late sign language exposure does not modulate the relation between spatial language and spatial memory in deaf children and adults

**DOI:** 10.3758/s13421-022-01281-7

**Published:** 2022-03-17

**Authors:** Dilay Z. Karadöller, Beyza Sümer, Ercenur Ünal, Aslı Özyürek

**Affiliations:** 1 Max Planck Institute for Psycholinguistics, Wundtlaan 1, 6525XD, Nijmegen, The Netherlands; 2 Centre for Language Studies, Radboud University, Nijmegen, The Netherlands; 3 Department of Linguistics, University of Amsterdam, Amsterdam, The Netherlands; 4 Department of Psychology, Ozyegin University, Istanbul, Turkey; 5 Donders Institute for Brain, Cognition and Behavior, Radboud University, Nijmegen, The Netherlands

**Keywords:** Spatial language, Spatial memory, Language and cognition, Sign language acquisition

## Abstract

Prior work with hearing children acquiring a spoken language as their first language shows that spatial language and cognition are related systems and spatial language use predicts spatial memory. Here, we further investigate the extent of this relationship in signing deaf children and adults and ask if late sign language exposure, as well as the frequency and the type of spatial language use that might be affected by late exposure, modulate subsequent memory for spatial relations. To do so, we compared spatial language and memory of 8-year-old late-signing children (after 2 years of exposure to a sign language at the school for the deaf) and late-signing adults to their native-signing counterparts. We elicited picture descriptions of Left-Right relations in Turkish Sign Language (*Türk İşaret Dili*) and measured the subsequent recognition memory accuracy of the described pictures. Results showed that late-signing adults and children were similar to their native-signing counterparts in how often they encoded the spatial relation. However, late-signing adults but not children differed from their native-signing counterparts in the type of spatial language they used. However, neither late sign language exposure nor the frequency and type of spatial language use modulated spatial memory accuracy. Therefore, even though late language exposure seems to influence the type of spatial language use, this does not predict subsequent memory for spatial relations. We discuss the implications of these findings based on the theories concerning the correspondence between spatial language and cognition as related or rather independent systems.

## Introduction

Children from early on communicate and reason about spatial relations. Prior work shows that there is a tight relation between these two systems and that children’s spatial language might predict their cognition such as their spatial memory (Abarbanell & Li, 2021; Dessalegn & Landau, 2008; Gentner, 2016; Gentner et al., 2013; Hermer-Vazquez et al., 2001; Karadöller, Sümer, Ünal, & Özyürek, 2021a; Landau, 2017; Loewenstein & Gentner, 2005; Miller et al., 2016). However, this evidence comes mostly from hearing children acquiring a conventional spoken language as their first language, who already have access to language from birth, and thus does not address whether being exposed to a language early or later in childhood can influence this relationship. This relationship can be studied by focusing on the language acquisition patterns of deaf children. The majority of deaf children (95%) are born to hearing parents and do not have immediate exposure to a conventional signed or a spoken language (Mitchell & Karchmer, 2004) – even with hearing aids or cochlear implants, which may not provide enough access to the surrounding speech (Hall et al., 2019; Henner & Robinson, 2021; Koulidobrova & Pichler, 2021). In such situations, many deaf children with hearing parents are exposed to a sign language later in life and mostly after entering a school for the deaf, especially in non-Western countries. Consequently, they are considered to be late signers because they lack immediate sign language exposure following birth. By contrast, deaf children with deaf parents are considered to be native signers, as they are exposed to a sign language from birth onwards by their caregivers.^1^

In this study, drawing evidence from deaf individuals who vary in the timing of exposure to a sign language we investigate whether late versus early exposure to a sign language (Turkish Sign Language, *Türk İşaret Dili*, TİD) predicts spatial language use as well as its relation to spatial memory. This allows us to investigate the relation between spatial language and memory in ways that would not have been possible by studying typically developing children who have immediate access to a spoken language.

Previous research has shown that preceding language exposure, deaf children of hearing parents in Turkey with no access to a conventional sign or spoken language lacked linguistic means to communicate about spatial relations and lagged behind hearing children acquiring a spoken language as their first language in a spatial memory task (Gentner et al., 2013). Further research showed that after 2 years of exposure to TİD, that is after starting the school for the deaf, another group of deaf children in Turkey was able to describe spatial relations as frequently as native-signing children using conventional linguistic forms (Karadöller, Sümer, & Özyürek, 2021b). However, the frequencies of different types of linguistic forms (i.e., classifier construction, relational lexemes, other forms such as pointing) used by this group of late signers differed significantly from that of native signers. Nevertheless, this previous research by Karadöller et al. (2021b) used a variety of spatial relations (i.e., locative relations such as Left-Right, In-On-Under) to elicit picture descriptions that were not balanced in terms of the number of stimuli items and tested relatively few participants (ten participants per group) that were not matched in terms of school experience or other non-linguistic cognitive skills. Furthermore, this study did not test whether late-signing children after 2 years of sign language exposure had comparable spatial memory performance to their native-signing peers in relation to their linguistic performance.

Therefore, our first goal here was to offer further empirical evidence on differential effects of late versus early exposure on spatial language use in children and adults by addressing the limitations of prior work. To do so, we used a more controlled set of stimuli of locative relations and test a higher number of native and late signers who are matched in several criteria (e.g., cognitive skills, schooling experience, etc.). Our second goal was to investigate whether the effects of late versus early exposure to language extend beyond the domain of spatial language and predict spatial memory accuracy. We address these goals by comparing deaf children and adults who are late and native signers of TİD on the frequency and the type of locative forms used in picture descriptions as well as the subsequent memory accuracy of the pictures they described.

We focus on the linguistic encoding of Left-Right relations, which has been found to be both cognitively and linguistically challenging for children (Abarbanell & Li, 2021; Benton, 1959; Harris, 1972; Martin & Sera, 2006; Piaget, 1972; Rigal, 1994, 1996; Sümer, 2015; Sümer et al., 2014) and also seems to differ across late versus early exposure to a sign language in terms of the type of linguistic forms used to express such relations (Karadöller et al., 2021b). Furthermore, previous work has shown that there is a relationship between encoding Left-Right relations in language and subsequent memory of these relations across hearing children acquiring a spoken language as their first language and signing children acquiring a sign language as their first language (Karadöller et al., 2021a). Yet, late language acquisition might modulate this relationship possibly due to shorter experience with the language or use of different linguistic forms due to late exposure.

In the following sections, we first describe the linguistic encoding of spatial relations in sign languages in general and specifically for TİD followed by the development of linguistic encoding in the case of late and early language exposure. Later, we review the literature on the relation between spatial language and spatial memory. Based on this literature, we build our predictions regarding the relation between spatial language and spatial memory accuracy considering late sign language exposure, as well as the frequency and the type of language use, as factors possibly influencing this relationship.

### Spatial language use in sign languages

In sign languages, spatial relations between objects are most frequently encoded by classifier constructions (e.g., Emmorey, 2002; Manhardt et al., 2020; Perniss et al., 2015; Schembri, 2003; Sümer, 2015; Zwitserlood, 2012). These constructions encode the size and shape of the objects through handshape classifications and allow signers to position these handshapes to represent the relative locations of these objects on the signing space in an analogue way to the real space.

**Fig. 1:**
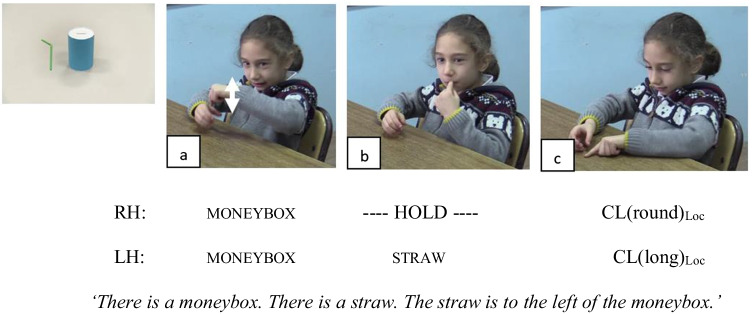
An example of a classifier construction by an 8-year-old native signer of TİD to encode the spatial relation between the moneybox and the straw

To illustrate, in order to describe the spatial relation between the objects in Fig. 1, the signer first introduces the lexical signs for the moneybox (Fig. 1a) and straw (Fig. 1b) and later chooses classifier handshapes that represent the size and shape of the moneybox (i.e., round handshape) and straw (i.e., elongated handshape). Next, she positions her hands on the signing space to represent the spatial relation between these objects in an analogue way to the real space (Fig. 1c). Thus, sign language encodings of space incorporate a visually motivated meaning mapping between the linguistic form and what it refers to in the real space (so-called *iconicity*). These are also known to be morphologically complex structures as the handshapes classify the size and shape of the objects located on the sign space (Zwitserlood, 2012).

Signers might also use other linguistic forms to convey spatial relations between objects such as tracing, relational lexemes, pointing, lexical verb placements (see Coding section and Figs. 4, 5 and 6 for more details). These forms are considered to be morphologically less complex than classifier constructions because they do not incorporate morphological handshape classification of objects (see Zwitserlood, 2012, 2021). Some of these forms include tracing of objects’ size and shape (see Fig. 4 in Coding section). Remaining strategies (i.e., relational lexemes, pointing, lexical verb placement) lack size and shape information about the objects (see Figs. 5 and 6 in the Coding section; see also Karadöller et al., 2021b; Manhardt et al. 2020). Nevertheless, all of these linguistic forms convey iconic mapping of the spatial relation between the objects to signing space (Karadöller et al., 2021a).

One prior study found that the above-mentioned differences in iconicity across the different types of linguistic forms influenced deaf signers’ visual attention prior to their linguistic descriptions of pictures containing Left-Right relations (Manhardt et al., 2020). Thus, the type of linguistic form used to encode the spatial relation in picture descriptions offers a good medium to investigate the relation between spatial language and other domains of cognition such as spatial memory. It is possible that as visual attention of signers changes prior to producing these different linguistic forms, this might then influence their subsequent memory of these relations after the linguistic production.

### Spatial language use in native versus late sign language exposure

Sign language expressions seem to help native-signing children encode some spatial relations earlier than hearing children acquiring a spoken language as their first language (Sümer, 2015; Sümer et al., 2014). For instance, although learning to encode Left-Right is known to be challenging for hearing children acquiring a spoken language as their first language (Abarbanell & Li, 2021; Benton, 1959; Harris, 1972; Piaget, 1972, Rigal, 1994, 1996), native-signing children are found to encode Left-Right relations between objects earlier (around age 5 years) in TİD than same-aged hearing children acquiring Turkish as their first language (Sümer, 2015; Sümer et al., 2014). The earlier encoding of Left-Right relations by native-signing children, albeit the morphologically complex nature of these forms, was attributed to the iconic nature of sign language encodings signed on the signing space or producing them on the left or right side of the body (see Karadöller et al., 2021b; Sümer, 2015; Sümer et al., 2014 for TİD; Manhardt et al., 2020, for Sign Language of the Netherlands; and see Fig. 5 in the Coding section for a body-anchored encoding of Left in TİD).

It is less well known how late exposure to language influences spatial language development. To our knowledge, only two studies investigated spatial language use in deaf children with hearing parents, who did not have immediate sign language exposure following birth (Gentner et al., 2013; Karadöller et al., 2021b). Gentner et al. (2013) compared spatial language use of 5-year-old deaf children before any exposure to a sign language to hearing children acquiring Turkish as their first language in a spatial language elicitation task containing descriptions of short video clips (e.g., toolbox moves on top of a school bus). Even though these children were not exposed to a conventional sign language, they have been reported to use gestural communication systems with their parents (i.e., homesign; see Goldin-Meadow, 2013) and they may also engage in fluid communication practices with others (see Moriarty Harrelson, 2019, and Henner & Robinson, 2021). However, results showed that these gestures or communication practices did not lead children to develop particular linguistic strategies that have been used in sign languages to convey spatial information to describe the video clips. This study, however, did not investigate how linguistic strategies of these children change after short-term exposure to a sign language at the school for the deaf.

A recent study showed that after 2 years of exposure to a sign language at the school for the deaf, late-signing children encoded spatial relations between objects equally frequently to native-signing children (Karadöller et al., 2021b). However, they differed from native-signing children in terms of the type of linguistic forms used in these encodings when describing Left-Right relations but not In-On-Under. Specifically, late-signing children used fewer classifier constructions in their descriptions and instead frequently used other morphologically simpler forms (i.e., pointing, tracing, and lexical verb placements). This was attributed to Left-Right relations being cognitively challenging compared to In-On-Under. This study also showed that the effects of late sign-language exposure persisted into adulthood. Late-signing adults also used fewer classifier constructions than their native-signing counterparts preferring other simpler forms to encode Left-Right relations albeit both groups encoded the spatial relation equally frequently. Other studies presented converging evidence for lingering problems to use classifier constructions by late-signing adolescents (Morford, 2003) and late-signing adults (Newport, 1988, 1990) who preferred morphologically simpler forms in the domain of motion event expressions.

Taken together, these studies showed that even though iconic linguistic forms that were signed on the space or on the coordinates of the body help native-signing children encode spatial relations in expressing Left-Right relations earlier than their hearing counterparts who acquire a spoken language as their first language (Sümer, 2015), this facilitating effect of iconicity does not provide an advantage in late sign language exposure. Late-signing children and adults prefer morphologically simpler forms (e.g., pointing) compared to classifier constructions – especially when encoding cognitively challenging spatial relations such as Left-Right (Karadöller et al., 2021b). To our knowledge, there is no information on whether late sign language exposure, as well as differences in language use due to late sign language exposure in encoding Left-Right relations influence other cognitive domains such as spatial memory.

### Relation between spatial language and spatial memory

Several studies reported that knowledge and use of spatial terms are important predictors of hearing children’s spatial reasoning in general and memory in particular (Casasola et al., 2020; Dessalegn & Landau, 2008; Gentner et al., 2013; Hermer-Vazquez et al., 2001; Loewenstein & Gentner, 2005; Miller et al., 2016; Pruden, et al., 2011; Shusterman et al., 2011; Simms & Gentner, 2019; Turan et al., 2021; Hermer-Vazquez et al., 2001). For example, using the term *middle* was related to better spatial skills measured by tasks involving the use of these terms (Simms & Gentner, 2019). Moreover, providing children with spatial terms before spatial memory tasks increased their accuracy (Loewenstein and Gentner, 2005; Miller et al., 2016). In Loewenstein and Gentner (2005), children were presented with two boxes (hiding box and finding box) that contain a card in each tier. First, children watched the experimenter placing a card in the hiding box and then were asked to find the card in the finding box. The card was always in the same spatial location (*top, middle, bottom*) in the two boxes. Prior to the task, half of the children played with the experimenter by using spatial terms (e.g., I am putting the winner *on* the box). The other half did not receive instructions that included spatial terms (e.g., I am putting the winner right here). Children who received instructions containing spatial terms outperformed the children who did not receive spatial terms when tested on the same day of the play session and even 2 days later. However, in this study, children were provided with spatial terms by the instructor. Hence, these findings leave open whether the use of spatial terms by children *themselves* predicts their memory accuracy.

All of the above studies focused on hearing children acquiring a conventional spoken language as their first language following birth. One prior study investigated whether or not being exposed to a conventional sign language predicts children’s performance in a spatial memory task (Gentner et al., 2013). In this study, Turkish deaf children who did not have a conventional language input were compared to same-age hearing children acquiring spoken Turkish as their first language using the same task as in Loewenstein and Gentner (2005) mentioned above. This time the task only contained instructions without spatial terms for hearing children acquiring a spoken language as their first language and for deaf children it contained pointing gestures to indicate locations as they did not have a conventional language. Results showed that deaf children without access to a conventional language did not have gestures resembling linguistic strategies used in sign languages to convey spatial relations and they performed significantly lower in a spatial memory task than hearing children and barely exceeded the chance level. This suggested a possible relation between spatial language use and spatial memory performance. However, one major drawback of this study was comparing deaf children with no language exposure to hearing children acquiring a spoken language as their first language where both deafness and language access differed between the groups.

In contrast to the evidence reviewed above suggesting a tight relation between spatial language and spatial memory, there are also claims that the relation between spatial language and memory may be nuanced or that these two systems are governed independently from one another (Li & Gleitman, 2002; Munnich et al., 2001; see also Gleitman and Papafragou, 2012, and Ünal & Papafragou, 2016, for reviews on the relation between language and cognition). These studies suggest that spatial memory performance may not be susceptible to (Li & Gleitman, 2002) or only partially depend on cross-linguistic variation in language use (Munnich et al., 2001). However, these studies focused only on hearing adults who acquired a spoken language as their first language and on spatial memory performance where language is not used to solve the task.

Based on prior work investigating the relation between spatial language and memory, there is an open question regarding the relation between spatial language and spatial memory in signing deaf children and whether late exposure to sign language modulates this relation (e.g., 2 years; see Karadöller et al., 2021b, for a similar approach). Another open question is whether the relation between spatial language and memory is predicted by the frequency and type of spatial language used by native- and late-signing children and adults.

## The present study

In order to fill the above-mentioned gaps in the literature on the effect of late language exposure on spatial memory accuracy, we compared spatial descriptions and spatial memory of late and native signers, in both children and adults. Our first goal was to offer further evidence on the effect of late sign language exposure on spatial language use across late and native signers by addressing limitations of prior work (Karadöller et al., 2021b). Our second goal was to investigate whether the effects of late versus early exposure extend beyond the domain of spatial language and predicted spatial memory. We also considered whether the frequency and the type of spatial language use predicted the relationship between spatial language and spatial memory given that they have been shown to differ in late exposure cases compared to early exposure and also differ in their morphological and iconic patterning.

Our empirical focus is the encoding of the Left-Right relations, which provides an excellent test bed as they have been found to be acquired differently by late and native signers (Karadöller et al., 2021b). The differences in linguistic forms to encode spatial relations by late and native signers might further modulate memory for spatial relations.

Participants saw displays with two objects in various spatial relations to each other and were asked to describe the target picture (pointed to by an arrow) to an addressee (see Karadöller et al., 2021a; Manhardt et al., 2020, 2021, for a similar procedure). Later, we tested their recognition memory accuracy of the target pictures in a surprise recognition memory task (see Karadöller et al., 2021a, for a similar procedure). We coded the spatial descriptions in terms of the frequency and the type of spatial language use and also calculated their recognition memory accuracy for the target pictures. This approach allowed us to test the relation between spatial language use and spatial memory accuracy in closely related tasks. Additionally, we measured participants’ visual-spatial working memory span via the computerized version of the Corsi Block Tapping Task in forward order to ensure similarities across late and native signers.

In this study, all late signers were exposed to a conventional sign language after starting the school for the deaf and late-signing children had 2 years of exposure to a sign language at the time of testing. Our motivation to test children after 2 years of exposure to a sign language was to replicate prior work that aimed to allow children to get enough exposure to acquire basic structures of the language as well as to ensure children have a cognitive readiness to express Left-Right spatial relations that were found to be acquired late by hearing children acquiring a spoken language as their first language (Abarbanell & Li, 2021; Benton, 1959; Harris, 1972; Piaget, 1972; Rigal, 1994, 1996; Sümer, 2015; Sümer et al., 2014).

### Predictions

Concerning spatial language use, based on previous work (Karadöller et al., 2021b), we predicted that late signers would have spatial encodings equally frequently to native signers for both children and adults. Moreover, we expected late signers, both children and adults, to use fewer classifier constructions compared to their native-signing counterparts.

Concerning spatial memory, we investigated whether late sign language exposure, as well as the frequency and the type of spatial language use, predicts spatial memory accuracy. One possibility is that these factors independently or interactively predict memory (e.g., Dessalegn and Landau, 2008; Gentner, 2016). If this were the case, we might obtain the following results:

First of all, late exposure, in general, might predict lower memory performance compared to early exposure possibly due to shorter experience with a conventional language. Specifically considering findings from children with no language exposure that showed impaired spatial memory compared to hearing children acquiring a spoken language as their first language (Gentner et al., 2013), 2 years of sign language exposure might not be enough for late-signing children to perform well in spatial memory tasks in comparison to their native-signing peers.

In addition, the frequency and the type of language might predict spatial memory based on previous research that has found relations between the two in spoken language research. For instance, encoding the spatial relation between the objects might predict better memory compared to when spatial relation was not encoded. Also, the type of the linguistic strategy such as encoding both the objects’ shape information and the information regarding the spatial relation between the objects (as in the case of classifier constructions and tracing) might predict better spatial memory compared to linguistic strategies that encode only the spatial information (as in relational lexemes, pointing, and lexical verb placements). Lastly, late sign language exposure might interact with the frequency and/or the type of spatial language use in predicting memory.

Alternatively, it is also possible that neither late sign language exposure nor frequency and type of language use predicts spatial memory. Such a finding would be in line with claims that the relation between spatial language and memory may be nuanced or that these two systems are governed independently from one another (Li & Gleitman, 2002; Munnich et al., 2001; see also Gleitman and Papafragou, 2012; Ünal & Papafragou, 2016).

## Method

The methods reported in this study have been approved by the Ethics Review Board of the Radboud University, Nijmegen, The Netherlands and Survey and Research Commission of the Republic of Turkey Ministry of National Education, Turkey.

### Participants

Profoundly deaf late-signing children (*n* = 23, nine females, *M*_*age*_ = 8; 6 years age range = 7; 3–9; 11), late-signing adults (*n* = 23, ten females, *M*_*age*_ = 36 years, age range = 25; 1–50; 1), native-signing children (*n* = 21, 12 females, *M*_*age*_ *=* 8; 5 years, age range = 6; 8–11), and native-signing adults (*n* = 26, 21 females, *M*_*age*_ = 29 years, age range = 18; 2–48; 7) of TİD participated in this study. Data from 12 additional children and seven additional adults were excluded for various reasons: failure to follow the instructions (n = 8), problems with the testing equipment (n = 6), and disruption during the testing sessions (n = 5). Participation was voluntary. Adult participants were given monetary compensation, child participants were given a gender-neutral color pencil kit. See appendices for information about handedness and the use of hearing devices across participants.

We determined the sample size based on convenience. Working with special populations poses certain challenges in reaching participants. Here, we report data from native signers who had been exposed to a sign language from birth by their signing deaf parents. This group represents 5% of the deaf population in the world (Mitchell & Karchmer, 2004) and in Turkey (İlkbaşaran, 2015). Hence, the number of participants in each group reported in this study was determined based on the total number of native-signing children attending the deaf schools in İstanbul that we could collect data from. We collected data from all students from these schools who matched our criteria (e.g., age, absence of comorbid health issues). Finally, to our knowledge, the current sample incorporates the largest number of native signers in comparison to previous studies conducted in the field.

All of the deaf children were recruited from the same schools and thus both late- and native-signing children were matched in terms of schooling experience. We compared two groups of children and adults to each other in terms of Age and Visual-Spatial Working Memory Span (i.e., Corsi Block Tapping Task) to make sure they were similar and there were no possible confounds for the measures we were interested in. We did these comparisons through Bayesian t-tests that tested for the probability of the mean difference (*M*_*DIFF*_) greater than zero and less than zero using the R package BayesianFirstAid (version 0.1; Bååth, 2014). Children were similar in Visual Working Memory (Bayesian two-sample t-test: *M*_*DIFF*_ (-5) > 0: *p* = 0.249, *M*_*DIFF*_ (5) < 0: *p* = 0.751) and in Age (Bayesian two-sample t-test: *M*_*DIFF*_ (-5) > 0: *p* = 0.293, *M*_*DIFF*_ (5) < 0: *p* = 0.707). Adults were similar in Visual Working Memory (Bayesian two-sample t-test: *M*_*DIFF*_ (-5) > 0: *p* = 0.728, *M*_*DIFF*_ (5) < 0: *p* = 0.272) but not in Age (Bayesian two-sample t-test: *M*_*DIFF*_ (-5) > 0: *p* = 0.006, *M*_*DIFF*_ (5) < 0: *p* = 0.944). Late-signing adults had slightly higher mean age than their native-signing counterparts.

### Materials

#### Description and familiarization tasks

**Fig. 2:**
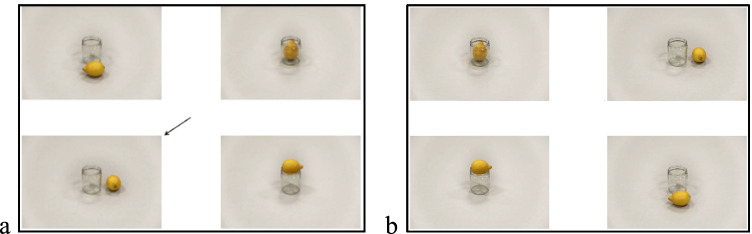
Example displays from the description (**a**) and memory tasks (**b**)

Stimuli consisted of 84 displays with four pictures (2 × 2 grid) showing the same two Figure (e.g., Pen) and Ground (e.g., Paper) objects in various spatial relations (i.e., Left, Right, Front, Behind, In, On) to each other (Fig. 2a). Ground objects were always located in the center of the pictures. Figure objects were positioned in relation to the ground objects. In each display, one of the pictures was the *target picture* to be described to an addressee sitting across the participant. Target pictures were indicated by an arrow appearing in the middle of the displays. Twenty-eight of the displays were the experimental displays that had a Left-Right spatial configuration of the items (e.g., the pencil is to the *left* of the paper). Half of the experimental displays (i.e., Non-contrast displays) had only one picture with Left-Right spatial configuration, other pictures in the display depicted In, On, Front, or Behind configurations. The remaining half (i.e., Contrast displays) had two pictures in Left-Right spatial configuration (if the objects in the target picture showed Left spatial configuration, one another picture in the display showed Right spatial configuration or vice versa), other pictures again depicted In, On, Front, or Behind configurations. We included different types of displays to encourage participants to provide descriptions as informative as possible that distinguish the target picture among the other alternatives. Moreover, in an attempt to avoid explicit attention to the Left-Right spatial configurations, we used filler displays (n = 56). Filler displays consisted of target items showing Front (n = 14), Behind (n = 14), In (n = 14), and On (n = 14) spatial configurations between the objects.

All displays were piloted to ensure that both children and adults could identify and name the objects. Within all 84 displays, Figure objects (e.g., pen) were presented only once. Ground objects (e.g., cup) were presented four times but always paired with other Figure objects (e.g., cup-pencil, cup-egg, cup-fork, cup-chocolate). The same Ground objects were only presented twice in a row. Moreover, the same spatial configuration as a target picture was presented only twice in a row to avoid biases to one type of spatial relation. Display order and the locations of the pictures in each display were randomized across each participant. Finally, there were two sets of displays with the same Ground objects but with different Figure objects. All other configurations were similar across the two sets.

#### Memory task

Stimuli consisted of 54 of the displays presented in the description task. We determined the number of items in the memory task based on a pilot study.^2^ Displays used in the memory task were similar to the description task except for the presence of an arrow (Fig. 2b). Display order and the arrangement of four pictures within the displays were randomized for each participant.

### Procedure

The experiment consisted of Familiarization Task, Description Task, Distractor Task, Surprise Recognition Memory Task, and Corsi Block Tapping Task. Familiarization and the Description tasks were designed as part of an eye-tracking experiment, but for the purpose of this study, we only reported the description data. All experimentation (i.e., Familiarization Task, Description Task, Memory Task, Corsi Block Tapping Task, and Flanker Task for adults^3^) was administered via a Dell laptop with software Presentation NBS 16.4 (Neurobehavioral Systems, Albany, CA, USA). No written instructions were used to avoid misunderstandings by signing participants. Instructions for all tasks were administered in TİD by a deaf research assistant who is a native signer. She was trained to use the same instruction across each testing session. For this training, we used a manual for testing that includes instruction in written and picture format. During the experiments, she used this manual as a guideline and administered the instructions similarly in each testing session. Note that there were occasional repetitions and variation when administering the instructions to children. The description task was video-recorded from the front and side-top angles to allow for sign coding.

#### Familiarization task

The purpose of this task was to acquaint participants with the general complexity of the displays with the 2 × 2 grid with two objects in various spatial configurations to each other. Participants were randomly presented with one of two sets of the displays as they received the other set in the description task.

#### Description task

**Fig. 3:**
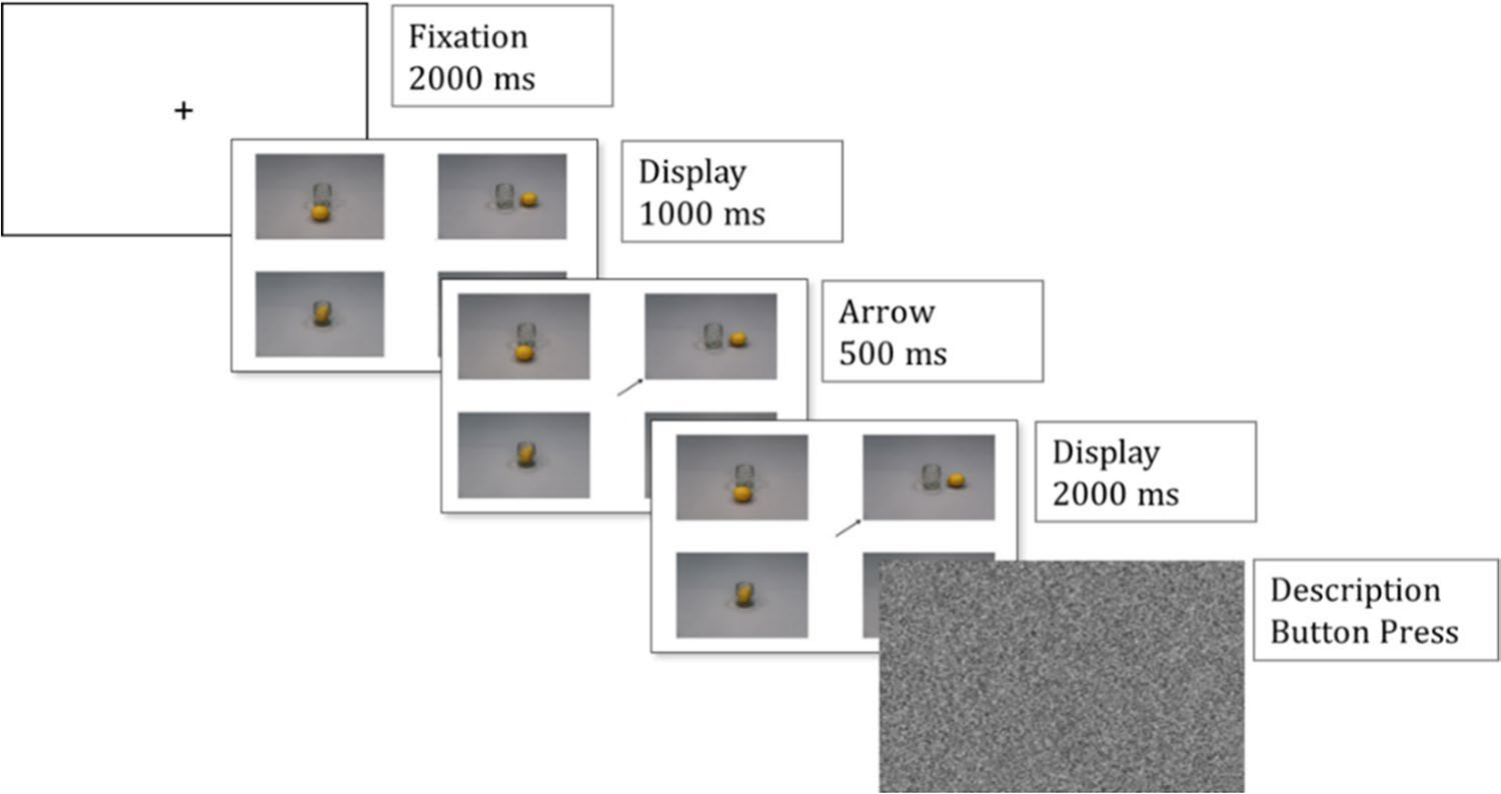
Timeline of a trial in the description task

Each trial started with a fixation cross (2,000 ms), followed by a display of four pictures (1,000 ms). An arrow was presented to specify the target item to be described for 500 ms. Later, four pictures remained on the screen for 2,000 ms more. Finally, a screen with visual white noise appeared that indicated participants to describe the target picture to a confederate addressee sitting across the table. Participants were informed that the addressee had the same displays without the arrow and with pictures in random locations. Upon the participant’s description, the addressee pretended to choose the described picture on her tablet. Participants were instructed to press the “Enter” key after the addressee selected the picture. Figure 3 illustrated the timeline of a trial in the description task.

Participants performed three practice trials at the beginning of the description task. These trials were repeated if necessary. Moreover, the experimenter also repeated the instructions in cases where participants did not understand the task instructions. The addressee did not provide feedback with regard to the correctness of the description to eliminate biasing the responses in the upcoming trials. In cases where participants’ descriptions included no spatial relation among the objects, the addressee only asked the location of the Figure object. In these instances, the addressee only asked for the location of the Figure object using the lexical sign of where and the lexical sign of the Figure object found in the target picture in TİD. No other instructions were given to the participants to ensure the consistency of the feedback.

The addressees were signers of TİD who had been exposed to sign language from birth by their deaf signing parents. The rationale for having a confederate addressee was to ensure consistency across sessions especially for children considering the previous reports on children’s tendencies to be under-informative in the presence of an inattentive addressee or in the absence of an addressee (Bahtiyar & Küntay, 2009; Girbau, 2001; Grigoroglou & Papafragou, 2019).

#### Distractor task

We used the Flanker Task (Eriksen & Eriksen, 1974) as a distractor between the Description and Surprise Memory Tasks to avoid memory effects due to recency. For adults, we used the original version of the Flanker Task, for children we used the child-friendly version with colored fish.

#### Memory task

Participants received the recognition memory task as a surprise in order to avoid possible effects on their linguistic production. Instructions were to click on the pictures that the participants’ described in the *Description task*. Participants received the trials one by one. New trials started as participants clicked on the pictures to make a selection. Participants completed the task at their own pace. That is why we were not interested in the reaction time of the responses, we were only interested in accuracy.

#### Visual working memory task

Participants received the computerized version of the Corsi Block Tapping Task to ensure that children and adults who had different timelines of language exposure have similar visual-spatial working memory spans (Corsi, 1972). This was especially important in looking back at studies showing mixed evidence for the differences in working memory across the late and native signers (see Marshall et al., 2015; Emmorey, 2002).

### Coding: Spatial descriptions

All sign descriptions were annotated and coded for the Target Pictures using ELAN (Version 4.9.3), a free annotation tool (http://tla.mpi.nl/tools/tla-tools/elan/) for multimedia resources developed by the Max Planck Institute for Psycholinguistics, The Language Archive, Nijmegen, The Netherlands (Wittenburg et al., 2006). Data were annotated by a hearing L2 signer of TİD and coded by another hearing L2 signer of TİD. Later, annotations and coding were checked by a trained native deaf signer of TİD. We did not have a reliability coding as we only included the linguistic forms that were unambiguously approved by this native signer in the final dataset.

First of all, we removed all “no attempt” trials, thus our coding is based on trials that participants attempted to describe the target picture. Further coding of the descriptions was performed in two steps. Descriptions were coded first for the presence of the spatial relation between the objects and next for the linguistic form that is used to localize the Figure object with respect to the Ground object. In some of the descriptions, participants only introduced the lexical signs for the objects but not the spatial relation between them or encoded an incorrect spatial relation between the objects (e.g., rather than locating the pencil to the left of the cup, participants located the pencil to the front of the cup). These descriptions were coded as not encoding a spatial relation. In a few other descriptions, participants used the addressee viewpoint instead of using the signer viewpoint. We did not eliminate descriptions that were signed from the addressee viewpoint and we counted them as having a spatial relation (for a similar strategy, see Karadöller et al., 2021a, b; Sümer, 2015).

**Fig. 4:**
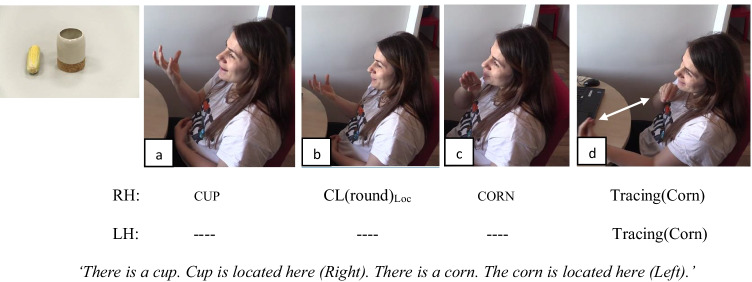
An example of Tracing strategy by a late-signing adult of TİD encoding the spatial relation between the cup and the corn

**Fig. 5:**
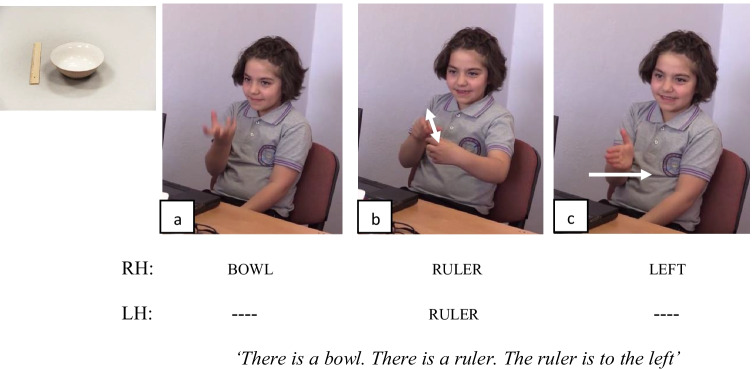
An example of a Relational Lexeme for the Left by a native-signing child of TİD encoding the spatial relation between the bowl and the ruler

**Fig. 6:**
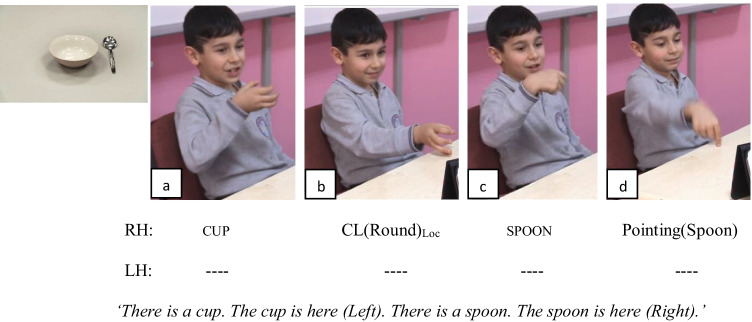
An example of Pointing strategy by a native-signing child of TİD encoding the spatial relation between the cup and the fork

Next, we identified five linguistic forms that encode spatial relations in sign languages. These forms were classifier constructions (Fig. 1) that were found to be the most frequent form to encode spatial relations in sign languages in general (see Emmorey, 2002) and specifically for TİD (Karadöller et al., 2021a, b; Sümer, 2015); tracing the Figure object’s size and shape on the signing space (Fig. 4) that has been reported in the literature as an alternative strategy that encodes both the object’s shape information as well as its relative loctaion (e.g., Perniss et al., 2015; Karadöller et al., 2021a, b; Sümer, 2015); relational lexemes (Fig. 5) that were used as the lexical signs for spatial terms in sign languages (see Arık, 2013; Karadöller et al., 2021b; Sümer, 2015, for TİD; and Manhardt et al., 2020, for Sign Language of the Netherlands); pointing with index finger or palm on a specific location on the signing space to indicate the location of the Figure object (Fig. 6; Karadöller et al., 2021b); lexical verb placements (Karadöller et al., 2021b; see also Newport, 1988, for a discussion of single morpheme signs).

We grouped the above-mentioned forms differently for production and memory analyses. For production analyses, we were interested in whether the frequency of classifier constructions (the morphologically complex forms that were found to be the most frequent form to encode space in sign languages; see Emmorey, 2002) differed from the Other simpler forms in terms of the frequency of use between native and late signers (Karadöller et al., 2021b; see Table 1 for the distribution of strategies within the Other forms). Some of the descriptions (total 10.06% of all data; 6.40% from late 3.62% from native signers) included the use of both classifier constructions and one of the Other forms. These cases were counted only for the presence of classifier construction to avoid double counting.

**Table 1:** Mean proportions (and Standard Errors of the Mean) for the type of linguistic forms used when spatial relation was expressed across Language Status and Age Group

	Classifier constructions	Other forms			
		Tracing	Relational lexemes	Pointing	Lexical verb placements
Adults					
Native	0.87 (0.007)	0.03 (0.003)	0.07 (0.010)	0.03 (0.005)	0.00 (0.000)
Late	0.64 (0.013)	0.10 (0.010)	0.11 (0.008)	0.14 (0.010)	0.01 (0.003)
Children					
Native	0.70 (0.019)	0.11 (0.011)	0.05 (0.008)	0.10 (0.012)	0.04 (0.007)
Late	0.54 (0.018)	0.21 (0.015)	0.06 (0.010)	0.17 (0.015)	0.02 (0.004)

For memory analyses, we grouped classifier constructions together with tracing, and the remaining forms were considered as Other forms. Again, some of the descriptions (total 5.98% of all data; 2.70% from late 3.28% from native signers) included the use of both classifier constructions or tracing and one of the Other forms. These cases were counted only for the presence of classifier constructions or tracing to avoid double counting. In this way, we could investigate whether linguistic forms that encode objects’ shape information and spatial information (i.e., classifier constructions and tracing) predicted memory accuracy differently than that of linguistic forms that only encode spatial information (remaining Other forms).

## Results

Data were analyzed using generalized binomial linear mixed-effects modeling (*glmer*) with random intercepts for Subjects and Items. All models were fit with the *lme4* package (version 1.1.17; Bates et al., 2014) in R (R Core Team, 2018). This mixed-effects approach allowed us to take into account the random variability due to having different participants and different items. We did not include random slopes in any of the models for two reasons. First, it was not possible to add random slopes to language production models as they were testing a between-subjects effect. Second, we did not add random slopes to memory models because doing so did not increase the model fit.

### Spatial language use

#### The frequency and type of spatial encoding

**Fig. 7:**
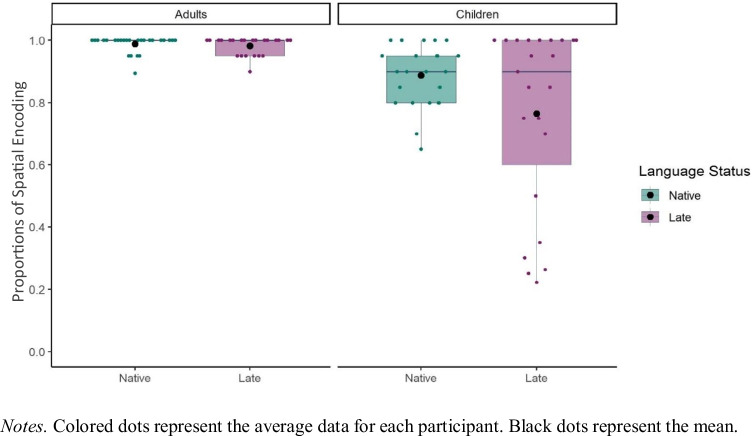
Proportions for the presence of Spatial Encoding in relation to Language Status and Age Group

First, we compared the frequency of descriptions that included spatial encoding between the objects to test whether late sign language exposure affected the amount of spatial encoding (Fig. 7). We used a *glmer* model to test the fixed effects of Language Status (Native, Late), Age Group (Adults, Children), and an interaction between Language Status and Age Group on the presence of Spatial Encoding (1 = Spatial Encoding, 0 = No Spatial Encoding) at the item level. All fixed effects (Language Status and Age Group) were analyzed with centered contrasts (0.5, -0.5). The model revealed a fixed effect of Age Group (*β* = 2.78, *SE* = 0.48, *p* < 0.001). Adults encoded spatial relations more frequently than children. There was no effect of Language Status (*β* = 0.29, *SE* = 0.57, *p* = 0.605) and no interaction between Age Group and Language Status (*β* = 0.70, *SE* = 0.97, *p* = 0.466). That is, averaged across Age Group, late signers encoded spatial relations equally frequently to their native signing counterparts.

**Fig. 8:**
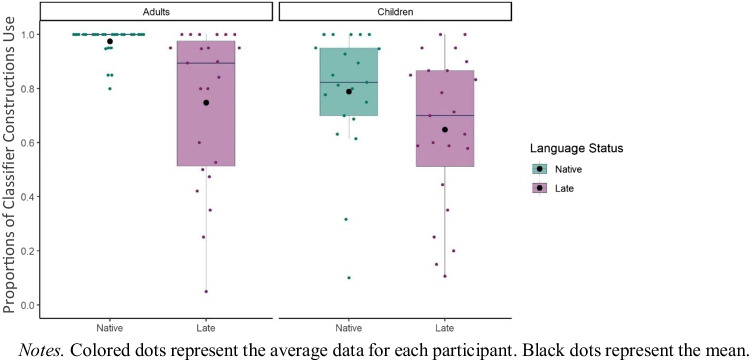
Proportions for the Presence of Classifier Constructions in relation to Language Status and Age Group

Next, we focused only on the descriptions that included spatial encoding and investigated to what extent late sign language exposure influenced the type of linguistic form used to encode spatial relation between the objects (Fig. 8; see also Table 1 for the distribution of linguistic forms). To do so, we compared the presence of classifier constructions across late and native signers. Here, descriptions, where classifier constructions were absent, included Other Forms (i.e., tracing, relational lexemes, pointing, lexical verb placements).

We used a *glmer* model to test the fixed effects of Language Status (Native, Late), Age Group (Adults, Children), and an interaction between Language Status and Age Group on the presence of Classifier Constructions (1 = Present, 0 = Absent) at the item level. All fixed effects (Language Status and Age Group) were analyzed with centered contrasts (0.5, -0.5). Model revealed a fixed effect of Age Group (*β* = 2.06, *SE* = 0.49, *p* < 0.001). Adults used classifier constructions more frequently than children. There was also a fixed effect of Language Status (*β* = -2.19, *SE* = 0.49, *p* < 0.001). Native signers used classifier constructions more frequently than late signers. Moreover, there was an interaction between Age Group and Language Status (*β* = -2.35, *SE* = 0.95, *p* = 0.01). To follow up on the interaction effect, we used *emmeans* package (Lenth et al., 2018; Searle et al., 1980). Separate comparisons for adults and children showed that late-signing adults used classifier constructions less frequently than native-signing adults (*β* = 3.36, *SE* = 0.76, *p* < 0.01). However, late-signing children used classifier constructions equally frequently to native-signing children (*β* = 1.01, *SE* = 0.63, *p* = 0.110).

### Spatial memory

In this section, we investigated whether late versus early exposure to language on the one hand and the frequency and type of spatial language on the other or their interaction predicted memory. First, we tested whether spatial memory was predicted by the presence of spatial encoding as well as by being a late versus a native signer. Secondly, we tested whether spatial memory was predicted by the type of language use as well as by being a late versus a native signer. We conducted our models separately for children and adults in order to avoid possible confounds affecting the memory performance due to age.

#### Spatial memory in relation to language status and the presence of spatial encoding

**Fig. 9:**
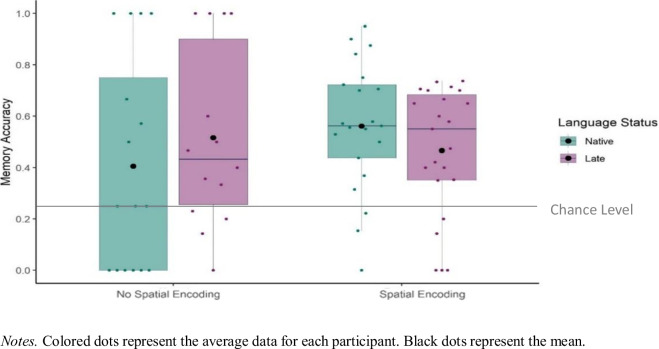
Mean proportions of Spatial Memory Accuracy of Children in relation to Language Status and the Presence of Spatial Encoding

Here, we did not report any comparisons for adults because spatial encoding was not suitable to be used as a fixed effect since both native- and late-signing adults encoded the spatial relation between the objects in 99% and 98% of the cases, respectively. For children, we investigated whether the spatial memory accuracy was predicted by the presence of spatial encoding and late sign language exposure for children (Fig. 9). We used a *glmer* model to test the fixed effects of Language Status (Native, Late), Spatial Encoding (Spatial Encoding, No Spatial Encoding), and an interaction between Language Status and Spatial Encoding on the Memory Accuracy (1 = Accurate, 0 = Not accurate) at the item level. All fixed effects (Language Status and Spatial Encoding) were analyzed with centered contrasts (0.5, -0.5). There was no effect of Language Status (*β* = -0.29, *SE* = 0.41, *p* = 0.479), Spatial Encoding (*β* = -0.42, *SE* = 0.25, *p* = 0.089), and no interaction between Language Status and Spatial Encoding (*β* = -0.41, *SE* = 0.50, *p* = 0.403). That is, for and children neither the Language Status of the participant nor the presence of Spatial Encoding predicted spatial memory accuracy.

#### Spatial memory in relation to language status and the type of language use

**Fig. 10:**
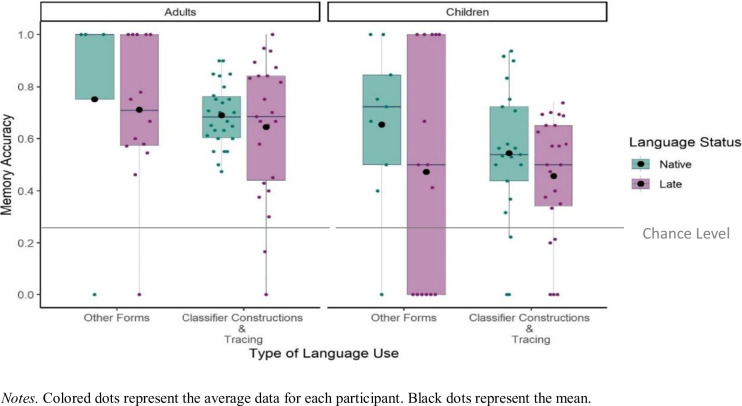
Mean proportions Memory Accuracy in relation to Language Status, Type of Language Use, and Age Group

Next, we focused only on the descriptions that included spatial encoding and investigated to what extent spatial memory was modulated by the type of language use and late sign language acquisition tested separately for children and adults (Fig. 10). For the Type of Language Use, we compared linguistic forms that encode both the objects’ shape information and spatial relation between the objects (i.e., classifier constructions and tracing) to forms that only encode spatial relation between the objects (i.e., relational lexemes, pointing, and lexical verb placements) to see if different types of linguistic forms predicted spatial memory.

We used separate *glmer* models for adults and children to test the fixed effects of Language Status (Native, Late), the Type of Language Use (Classifier Construction and Tracing, Other Forms), and the interaction between Language Status and the Type of Language Use on the presence of Memory Accuracy (1 = Accurate, 0 = Not accurate) at the item level. All fixed effects (Language Status and the Type of Language Use) were analyzed with centered contrasts (0.5, -0.5). There was no effect of Language Status (*Adults: β* = -0.75, *SE* = 0.60, *p* = 0.208; *Children: β* = -0.62, *SE* = 0.43, *p* = 0.154), Type of Language Use (*Adults: β* = -0.65, *SE* = 0.57, *p* = 0.253; *Children: β* = -0.39, *SE* = 0.32, *p* = 0.233), and no interaction between Language Status and the Type of Language Use (*Adults: β* = 1.49, *SE* = 1.14, *p* = 0.190; *Children: β* = 0.45, *SE* = 0.64, *p* = 0.487). That is, for both adults and children, neither the Language Status of the participant nor the Type of Language use predicted Spatial Memory Accuracy.

## Discussion

In this study, we aimed to provide evidence for the relation between spatial language and memory and asked whether and how late or early language exposure predicts the relation between spatial language use and spatial memory in deaf signing children and adults by also taking into account the linguistic productions of both language exposure groups. Going beyond previous work (Karadöller et al., 2021b), first, we tested whether the effects of late language exposure on the frequency and the type of spatial language use in encoding Left-Right relations obtained for children and adults were reproducible using a more balanced stimuli set and a larger sample while controlling for general cognitive skills across the groups. Secondly, we investigated whether late sign language exposure and the possible differences in the frequency and the type of spatial language use predicted the relationship between spatial language and memory.

Concerning spatial language use, our results showed that late language exposure did not affect the frequency of spatial language use between both late- and native-signing children and late- and native-signing adults. However, late language exposure affected the type of spatial language use where late signers used fewer classifier constructions compared to native signers. This was also modulated by age group. That is, late-signing adults used fewer classifier constructions than native-signing adults, whereas late- and native-signing children used classifier constructions equally frequently. Moreover, overall adults encoded the spatial relation between the objects more frequently and used classifier constructions more frequently than children. Finally, we found that neither late language exposure nor the frequency and the type of language use or their interaction predicted memory accuracy.

### Effects of late sign language exposure on the frequency and the type of spatial language use

Our results were mostly in line with previous findings for the effects of late sign language exposure on the frequency and the type of spatial language use, replicating Karadöller et al. (2021b), except for the use of classifier constructions for children. Similar to Karadöller et al. (2021b), we found that, after 2 years of sign-language exposure, late-signing children could encode spatial relation between the objects equally frequently to their native-signing peers, and this effect persisted into adulthood. Here, we extend the literature on the spatial language development of deaf children with hearing parents by demonstrating that having a short-term exposure to language could scaffold the development of linguistic encoding of space.

Moreover, similar to previous work (Karadöller et al., 2021b), we found that adults encoded spatial relations more frequently than children regardless of language exposure. This shows that even by age 8 years, neither native- nor late-signing children are adult-like in the frequency of descriptions that encode Left-Right relations. With this finding, we contribute to claims pointing to the challenge of spatial domain in linguistic development specifically for Left-Right – albeit they are learned earlier by signing children compared to hearing children acquiring a spoken language as their first language (Abarbanell & Li, 2021; Benton, 1959; Harris, 1972; Piaget, 1972; Rigal, 1994, 1996; Sümer, 2015; Sümer et al., 2014).

Secondly, with regard to the type of spatial language use, unlike prior work showing that both late-signing children and adults used classifier constructions less frequently than their native-signing counterparts (Karadöller et al., 2021b), in the current study, we found that this effect was modulated by age. That is, late-signing adults used classifier constructions less than their native-signing counterparts, however, late-signing children used classifier constructions equally frequently to their native-signing peers. Considering that the current study incorporated more stimuli materials that were controlled in many aspects, including a larger sample of participants who were matched in several criteria such as general cognitive abilities, we believe it may better represent the effect of late sign language acquisition on spatial language use. That is, the effect of late sign language exposure may be more salient for adults than for children. This could be partially due to the fact that native-signing children are also not adult-like in encoding Left-Right relations and thus differences between late- and native-signing children are less visible. This provides more evidence for the claim that the effect of late sign language exposure on spatial language use is more strongly observed as signers get older (see Morford, 2003; Newport 1988, 1990). Overall, the low frequency of classifier constructions used by late-signing adults compared to their native-signing counterparts might point out a general linguistic challenge for late signers to acquire morphologically complex forms (see Newport, 1990) especially in encoding Left-Right relations despite their iconicity (Karadöller et al., 2021b). Thus, the facilitating effect of iconicity of the linguistic forms could only be observed in the early exposure cases.

### Relation between spatial language use and spatial memory accuracy

Our study showed that late sign language exposure did not predict spatial memory performance either for children or for adults. Furthermore, we found that neither the frequency nor the type of spatial language use predicted spatial memory accuracy. We also did not detect any interaction between late sign language exposure and the frequency or the type of spatial language use.

Specifically, for the type of spatial language use, we could have expected to see linguistic forms that encode both the objects’ shape information and the spatial relation to predict better memory compared to linguistic forms that only encode spatial information. This was based on previous research showing that encoding both the objects’ shape information and the spatial relation between the objects recruited visual attention differently during planning of a linguistic description than that of linguistic forms that only encoded the spatial relation between the objects (Manhardt et al., 2020). This result may suggest that the influence of spatial language on cognitive processes may emerge during language use such as in visual attention while planning linguistic descriptions, but such an effect may not always last after language use is completed.

It is also possible that our paradigm could not detect the differences in spatial memory. For example, we did not ask participants to respond quickly in the memory task and this might have diminished the differences in memory accuracy as participants might have taken their time to find the correct answer. Moreover, it is also possible that having used pictures as the stimuli material (*picture superiority effect*; Paivio et al., 1968) coupled with asking participants to describe the target pictures during encoding (*production effect*; Conway & Gathercole, 1987) might have boosted memory accuracy (see Zormpa et al., 2019, for the complex interplay between picture superiority and production effects in psycholinguistics research) and removed the possible effects.

Therefore, our findings, in general, do not support previous findings showing a relation between spatial language use and later memory accuracy (Abarbanell & Li, 2021; Casasola et al., 2020; Dessalegn & Landau, 2008; Gentner, et al., 2013; Hermer-Vazquez et al., 2001; Loewenstein & Gentner, 2005; Miller et al., 2016; Pruden, et al., 2011; Shusterman et al., 2011; Simms & Gentner, 2019). Rather, our findings are more in line with the claims that relation between spatial language and memory might be nuanced and the two systems are governed independent of each other (e.g., Gleitman and Papafragou, 2012; Landau, 2017; Landau & Jackendoff, 1993; Li & Gleitman, 2002; Munnich et al., 2001; Ünal & Papafragou, 2016). Accordingly, our findings might provide empirical support to the argument that spatial concepts, such as spatial memory in our case, may not be enhanced through language, but develop independently of the experience with language. We believe the structure of our design providing a one-to-one correspondence between the linguistic production and memory, where we measured the recognition memory accuracy of the *same* pictures that participants had described and as a function of their particular productions, strengthens this conclusion. Nevertheless, in order to provide a complete picture of linguistic and cognitive development of space in late sign language exposure cases, further research should concentrate on longitudinally studying the same children before and after language exposure and comparing them to native-signing children to more precisely estimate the effects of late exposure on memory.

## Conclusion

Here, we showed that late exposure to language influences the frequency and the type of spatial language use for encoding Left-Right relations, but it does not necessarily predict the relation between spatial language and memory – even when the frequency and the type of language use are taken into account (Gentner et al., 2013; Hyde et al., 2011; Karadöller et al., 2021b). Even though late exposure affects the use of morphologically complex forms to encode spatial relations (e.g., classifier constructions), albeit more visible in adults than children, this does not, in turn, predict memory performance even when the level of iconicity in the linguistic forms are taken into account. These findings are in line with literature showing that the development of spatial language and spatial memory might be rather independent of each other (Gleitman and Papafragou, 2012; Li & Gleitman, 2002; Munnich et al., 2001; Ünal & Papafragou, 2016).

## Appendix 1

Handedness of the participants

Numbers in the graphs represent the number of participants in each category. Numbers add up to the total number of participants in a given participant group.

## Appendix 2

Use of hearing devices

The numbers in the graphs represent the number of participants in each category. Numbers add up to the total number of participants in a given participant group.

*Notes.* As teacher reports and checks by the experimenter showed, the deaf children were unable to respond when they were called by their names in the auditory modality despite some of them using hearing aids. This suggests that hearing aids did not provide optimal hearing outcomes.

## Footnotes

1 Following several scholars (e.g., Lillo-Martin & Henner, 2021; Mayberry, 1998; Newport, 1990), we use the term *native* to refer to deaf individuals who have a sign language exposure immediately following birth from their signing deaf parents. We use the term *late* to refer deaf individuals who have a sign language exposure mostly at the school for the deaf from their deaf signing peers. All of our late as well native-signing participants started the school for the deaf after age 6 years (see İlkbaşaran, 2015, for the Turkish context). In the schools for the deaf in Turkey, Turkish Sign Language is not part of the official curriculum, but is mainly spread through deaf peers to newcomers.

2 Initially, we piloted the memory task with the full set of displays (N = 84) used in the description task. This resulted in floor levels of memory accuracy for children. Next, we piloted the memory task with half of the displays in the description task (n = 42). This, however, resulted in ceiling performance for adults. Finally, we optimized the number of displays to 54, which included 20 target pictures. Pilot tests showed no ceiling or floor performance for adults and/or children.

3 Children received the Flanker task on the same laptop but with E-prime software.
